# Prediction of RECRUITment In randomized clinical Trials (RECRUIT-IT)—rationale and design for an international collaborative study

**DOI:** 10.1186/s13063-020-04666-8

**Published:** 2020-08-21

**Authors:** Benjamin Kasenda, Junhao Liu, Yu Jiang, Byron Gajewski, Cen Wu, Erik von Elm, Stefan Schandelmaier, Giusi Moffa, Sven Trelle, Andreas Michael Schmitt, Amanda K. Herbrand, Viktoria Gloy, Benjamin Speich, Sally Hopewell, Lars G. Hemkens, Constantin Sluka, Kris McGill, Maureen Meade, Deborah Cook, Francois Lamontagne, Jean-Marc Tréluyer, Anna-Bettina Haidich, John P. A. Ioannidis, Shaun Treweek, Matthias Briel

**Affiliations:** 1 Basel Institute for Clinical Epidemiology and Biostatistics, Department of Clinical Research, University Hospital Basel and University of Basel, Basel, Switzerland; 2 Department of Medical Oncology, University Hospital and University of Basel, Basel, Switzerland; 3 Department of Biostatistics & Data Science, University of Kansas Medical Center, Kansas City, KS 66160 USA; 4 University of Kansas Cancer Center, University of Kansas Medical Center, Kansas City, USA; 5 Division of Epidemiology, Biostatistics, and Environmental Health, School of Public Health, University of Memphis, Memphis, TN 38152 USA; 6 Department of Statistics, Kansas State University, Manhattan, KS 66506 USA; 7 Cochrane Switzerland, Center for Primary Care and Public Health (Unisanté), University of Lausanne, Lausanne, Switzerland; 8 Department of Health Research Methods, Evidence, and Impact, McMaster University, Hamilton, Canada; 9 Department of Mathematics and Computer Science, University of Basel, Basel, Switzerland; 10 CTU Bern, University of Bern, Bern, Switzerland; 11 Centre for Statistics in Medicine, Nuffield Department of Orthopaedics, Rheumatology and Musculoskeletal Sciences, University of Oxford, Oxford, UK; 12 Clinical Trial Unit, Department of Clinical Research, University Hospital Basel and University of Basel, Basel, Switzerland; 13 Nursing, Midwifery, and Allied Health Professionals Research Unit, Glasgow Caledonian University, Glasgow, UK; 14 Centre de recherche du CHU de Sherbrooke and Université de Sherbrooke, Sherbrooke, Canada; 15 Assistance Publique-Hopitaux de Paris, Hopitaux Universitaires Paris Centre, Unité de Recherche clinique, 27 rue du Faubourg Saint-Jacques, 75014 Paris, France; 16 Department of Hygiene, Social-Preventive Medicine & Medical Statistics, School of Medicine, Faculty of Health Sciences, Aristotle University of Thessaloniki, Thessaloniki, Greece; 17 Meta-Research Innovation Center at Stanford (METRICS) and Departments of Medicine, of Health Research and Policy, of Biomedical Data Science, and of Statistics, Stanford University, Stanford, USA; 18 Health Services Research Unit, University of Aberdeen, Aberdeen, UK

**Keywords:** Recruitment, Accrual, Prediction, Randomized clinical trials

## Abstract

**Background:**

Poor recruitment of patients is the predominant reason for early termination of randomized clinical trials (RCTs). Systematic empirical investigations and validation studies of existing recruitment models, however, are lacking. We aim to provide evidence-based guidance on how to predict and monitor recruitment of patients into RCTs. Our specific objectives are the following: (1) to establish a large sample of RCTs (target *n* = 300) with individual patient recruitment data from a large variety of RCTs, (2) to investigate participant recruitment patterns and study site recruitment patterns and their association with the overall recruitment process, (3) to investigate the validity of a freely available recruitment model, and (4) to develop a user-friendly tool to assist trial investigators in the planning and monitoring of the recruitment process.

**Methods:**

Eligible RCTs need to have completed the recruitment process, used a parallel group design, and investigated any healthcare intervention where participants had the free choice to participate. To establish the planned sample of RCTs, we will use our contacts to national and international RCT networks, clinical trial units, and individual trial investigators. From included RCTs, we will collect patient-level information (date of randomization), site-level information (date of trial site activation), and trial-level information (target sample size). We will examine recruitment patterns using recruitment trajectories and stratifications by RCT characteristics. We will investigate associations of early recruitment patterns with overall recruitment by correlation and multivariable regression. To examine the validity of a freely available Bayesian prediction model, we will compare model predictions to collected empirical data of included RCTs. Finally, we will user-test any promising tool using qualitative methods for further tool improvement.

**Discussion:**

This research will contribute to a better understanding of participant recruitment to RCTs, which could enhance efficiency and reduce the waste of resources in clinical research with a comprehensive, concerted, international effort.

## Background

Randomized clinical trials (RCTs) are considered standard to evaluate benefits and harms of healthcare interventions. RCTs are major undertakings that require enormous resources and coordination of multiple stakeholders. One out of four RCTs is prematurely discontinued because of poor recruitment of participants, other reasons suggesting futility, unexpected harm, early superiority, or administrative problems [1–4]. The leading reason for discontinuation is poor recruitment [5]. In particular, investigator-initiated RCTs with smaller sample size carry the highest risk for premature discontinuation due to poor patient recruitment. Such discontinued RCTs are also less likely to be published in medical journals [5].

Early discontinuation for poor recruitment is problematic: First, the pertinent research question typically remains unanswered. Second, precious resources for clinical research are wasted if costly RCTs need to be discontinued because of overoptimistic recruitment assumptions, in particular, if whatever results and lessons learned remain unpublished [6]. Third, public and patients’ trust and willingness to participate in clinical studies are compromised if RCTs are not professionally planned and conducted to minimize the risk for insufficient recruitment.

A central question for investigators during planning and conducting RCTs is: Given the expected number of participating centers and number of patients per center, how long will it take to recruit the target number of patients? Once the trial has started recruiting, the central question changes into: Given the recruitment data we have so far, how long will it take to recruit the target number of patients now, and do we have to adjust our initial estimates and plans?

Therefore, evidence-based guidance and tools to predict and monitor recruitment of patients to RCTs are needed [7]. Methodological research on recruitment to clinical trials has increasingly become of interest to clinical researchers; exemplary initiatives are the working group by the Medical Research Council (UK) [8], the Clinical Trials Transformation Initiative (CTTI) [9], and the Trial Forge-collaboration [10].

A recent systematic review on recruitment models identified 13 articles reporting on different recruitment models and provides a comprehensive exploration on recruitment topics from a statistical perspective [11]. Of note, while in some of the studies a validation of proposed models is reported, systematic empirical investigations and validation studies of proposed recruitment models with large datasets are still lacking.

One of the identified models was a Bayesian model developed by Jiang et al. [12] with an open source package for the statistical program R [13]. This model requires only input information that is readily available to trialists and trial coordinators, such as planned sample size, anticipated time needed for total trial recruitment, number of patients recruited to date, and interim time point (e.g., 6 months after trial initiation). Given the recruitment process until the interim time point, the program allows either calculating the time needed to recruit the remaining number of patients or predicting anticipated recruitment over a given period of time. The program provides a predicted time accompanied by a prediction interval and illustrative plots.

We initiated the international collaborative project on RECRUITment In Trials (RECRUIT-IT) to investigate performance of the Bayesian recruitment model developed by Jiang et al. [12] to provide evidence-based recommendations for the planning and monitoring of patient recruitment in RCTs.

We aim to accomplish this goal through four specific objectives:

1. To establish a large database with empirical individual patient recruitment data from a variety of RCTs.
2. To investigate overall participant recruitment patterns of RCTs and recruitment patterns at individual study sites and their association with the overall recruitment process.
3. To investigate the validity of the freely available Bayesian recruitment model [12] to predict participant recruitment into RCTs.
4. To refine the recruitment model from objective 3 or to develop a new model based on the findings from objectives 1–3 for an interactive and user-friendly tool to assist trial investigators in the planning and monitoring of the recruitment process.

## Methods/design

### Study design and eligibility criteria

This project will be based on empirical recruitment data from RCTs. We will include parallel group RCTs that have completed or terminated the recruitment process for all participating trial sites, evaluating any healthcare intervention where participants had the free choice to participate. Ideally, individual recruitment data (the date of randomization for each patient) are available; however, RCTs with recruitment data aggregated on a monthly basis will also be included. There will be no limitation with respect to sample size, medical field, intervention, jurisdiction, or funding source. However, we will exclude RCTs if they meet one of the following criteria, of which we assume that such studies are less attractive for participants than other trials, with inherent implications for recruitment patterns:

1. The trial investigated pharmacokinetics only.
2. The trial was actually a sub-study of a trial (e.g., whereby the main outcomes are biomarker studies).
3. The trial was a pilot trial (e.g., whereby the main outcomes are related to feasibility).

### Selection of RCTs and sample size

We aim to include RCTs from the following research groups that we approached through our personal networks: (i) AIDS Clinical Trials Group (ACTG) [14, 15], (ii) Paris Hospital Network (Assistance publique – Hôpitaux de Paris), (iii) Swiss Group for Clinical Cancer Research (SAKK), (iv) Johns Hopkins Medical Institutions, (v) clinical researchers from the university hospitals Basel and Bern (Switzerland), (vi) Canadian Critical Care Trials Group, and (vii) University of Kansas Cancer Center (KUCC) research group. Further networks are being contacted. In addition, we also plan to include RCTs via the clinicalstudydatarequest.com website.

Considering the abovementioned sources of RCTs, we estimate that individual patient recruitment data of at least 300 RCTs will be available for this project. This is a convenience sample. We aim to include as many RCTs from different fields of healthcare as possible.

### Data collection and research ethics

We will collect the following data (among other):

1. Patient-level: Date of randomization.
2. Site-level: Date when a trial site was activated and when it was closed (if available), country, total of recruited/randomized patients, number of eligible patients who declined to participate (if available), and whether the site is at the institution of the principal investigator (if available).
3. Trial-level (trial characteristics): Patient population, medical field, type of intervention (e.g., drug intervention), blinding status, target sample size (includes statistical sample size and overall recruitment goal [if available]), anticipated recruitment time, number of trial sites, involved countries, setting of recruitment (e.g., intensive care), primary endpoint, trial completion status (if discontinued, reason for the discontinuation), and funding sources. Information on trial characteristics is taken from the recruitment data source, trial protocols (preferred option if available), journal publications, or trial registries.

### Definitions

The “recruitment target” is the planned number of patients to be randomized as reported in the latest version of the respective RCT protocol; the primary journal publication, i.e., the one reporting the primary outcome results; or the latest update of the trial registry entry (in descending order in case of discrepancies). In case no target sample size, but target number of events is reported (e.g., in case of a time-to-event outcome), we will take the approximate number of patients needed if reported; otherwise, the RCT will be excluded from the analyses outlined below. We will record the calculated sample size as well as the recruitment target. This is to account for the fact that trial teams often inflate the calculated sample size to allow for some losses-to-follow-up, which means the statistically required sample size is typically smaller than the recruitment target.

“Recruitment time” is defined as the time (in days/months) from the date of the first patient randomized to the date of last patient randomized. Where available, we will also consider the time from trial activation until the date of last patient randomized to account for the possible gap between trial activation and first patient randomized.

“Anticipated recruitment time” is defined as the total recruitment time the investigators anticipated for completing patient recruitment at the planning stage before the RCT was initiated (i.e., before first patient randomized).

The “cumulative participant recruitment” is the participant total recruited over the recruitment time (absolute number) or the proportion of the target sample size recruited over the recruitment time.

We define “interim landmarks” based on the recruitment time and the proportion of target sample size (and recruitment target) recruited during the RCT recruitment time. For the interim landmarks based on time, we will use months 1, 3, 6, 9, and 12 after RCT initiation. For the landmarks based on the proportion of target sample size, we will use 10% intervals (e.g., 10%, 20%, or 30% of target sample size recruited).

We will consider an RCT to be prematurely discontinued if the investigators who provide individual recruitment data indicate recruitment discontinuation for one of the following or any other reasons:

1. Poor recruitment (less than 90% of the statistical sample size; 80% or less for sensitivity analysis)
2. Benefit
3. Futility (for reason other than poor recruitment)
4. External evidence
5. Harm
6. Other administrative reasons

If no direct contact to the investigators is possible, we will rely on published reports (e.g., journal publications, trial registries) to determine the reason for discontinuation. In case the achieved sample size is below 90% of the target sample size (or 80% for sensitivity analysis) and no reason is identifiable, we will categorize the RCT as discontinued and record “due to unknown reason.” All other RCTs not meeting abovementioned criteria will be considered completed.

### Analysis

We will summarize and present the characteristics of all included RCTs using descriptive statistics (means, medians, ranges, and frequencies or proportions as appropriate).

For each RCT, we will calculate the total recruitment time in months. Data on cumulative recruitment of participants will be presented by plotting the number of patients—expressed as proportion of the original recruitment target to facilitate comparison across trials—over time separately for each RCT; the resulting plots we will call recruitment trajectories. We will also provide overall averaged recruitment rates (total number of recruited patients/recruitment time [months]) for each RCT.

For the following analyses, we will mainly consider RCTs that have been completed or where recruitment has been discontinued due to poor recruitment. RCTs stopped for other reasons than poor recruitment will be included for additional sensitivity analyses.

We aim to examine the following (inspired by findings from Haidich and Ioannidis [14–16]): (1) associations between study site recruitment and speed of participant recruitment (e.g., recruitment trajectories for RCTs with more than 80% of recruiting sites ready to recruit participants within the first month versus those RCTs with less than 80%), (2) investigation of seasonal differences of participant recruitment rates [16], (3) associations between early recruitment trajectory slopes and overall recruitment success (i.e., achievement of more than 90% of the target sample size), and (4) investigation of how the recruitment time matches the anticipated recruitment time at the planning stage. However, based on our previous experience [5, 17], we assume that the anticipated time of patient recruitment will not be available for most RCTs (be it on protocol or journal publication level).

In further analyses, we will check whether the findings from Haidich and Ioannidis (i.e., recruitment slope of first 1–2 months of a trial is highly predictive of further patient recruitment) hold true for RCTs outside the AIDS Clinical Trials Group. For this, we will split the dataset into those RCTs used by Haidich and Ioannidis and all others. Specifically, we will investigate the following: (1) the extent to which speed of recruitment during the first 3 months will correlate with the proportion of RCTs for which the target sample size was attained; (2) the extent to which recruitment during the first month or two will correlate with subsequent recruitment; (3) the extent to which the acceleration of recruitment in the first 2 months (the ratio of second to first month accrual) was also related significantly to the eventual accrual; (4) whether the more patients were enrolled in the first 2 months, the greater the absolute enrolment and the proportion of target enrolment eventually achieved; (5) whether all trials that recruit < 30% of their target sample size after 10 months fail to reach their target eventually; and (6) whether no trials show marked acceleration of accrual over time.

Based on our systematic search and earlier reviews [7, 18], the Bayesian model developed by Jiang et al. [12] is the only model that comes with a freely available software package (written for R [www.r-project.org]) dedicated to predicting recruitment in clinical studies. We will therefore investigate the validity of this prediction model at baseline (before start of recruitment) and at the mentioned interim landmarks guided by the following questions:

- How large is the difference (in months) between the model estimate and the actual recruitment time?
- In how many cases does the 95% credible interval (of the predicted estimate) include the actual recruitment time?
- How do the accuracy and precision of prediction increase over time if model estimates are calculated at interim landmarks (e.g., months 1, 3, 6, 9, 12) or according to achieved recruitment (e.g., 10%, 20%, or 50% of target sample size)?
- How do different assumptions (e.g., informative or non-informative priors) influence prediction accuracy?
- In case of multicenter trials, how does the prediction change if the date of site activation is considered in such a prediction model?

Simple linear regression models following a frequentist approach are commonly used to predict the future recruitment process, which assume that the accrual rate is constant for each individual trial or individual trial sites. To investigate whether the more flexible Bayesian recruitment model, which takes into account information on accrual accumulated up to a monitoring point in the study in addition to including prior information from historical data, provides better estimates for recruitment prediction, we will compare the two models with respect to the following:

1. The accuracy of accrual prediction (number of participants, *n*) at expected trial duration time (*T*).
2. The accuracy of recruitment duration prediction (how long will the trial take, *T*) to reach the target sample size of *n* participants.

For the prediction of number of patients recruited at a specific time point, we will assume the information of early accrual to be known. Based on the information from the early recruitment frame, we will first calculate the accrual prediction using both methods (simple linear model versus Bayesian model). To quantify the respective model performance, we will calculate the percent error (%*Rbias*) statistic: %*Rbias* = |Predicted − True|/True × 100%*.*

Here, “Predicted” means the predicted value by the model and “True” means the actual number of patients recruited. We will calculate %*Rbias* at different time points. In addition, the %*Rbias* will be visually displayed over time. For example, we will compare the %*Rbias* of the two methods from 1 to 12 months of recruitment time for each RCT.

In addition, to predict the number of participants recruited, Bayesian methods can also provide a 95% prediction interval for the anticipated trial completion time, which can be used to determine whether a trial is slow or on-target with respect to accrual. The RCTs can be identified as having slow accrual if their anticipated completion time, *T*, is smaller than the lower bound of the prediction interval of completion time *T*_Pred_. Furthermore, the Bayesian model can identify studies with high risk of accrual failure by estimating the probability that a trial will achieve its accrual target late. The probability of being late by a given time (*P*_late_) offers an effective way of monitoring recruitment progress and facilitates decision-making for strategically allocating resources. For example, to estimate the risk for a trial, we can draw 50,000 Markov-Chain Monte-Carlo (MCMC) samples from the Bayesian posterior predictive distribution for *T*_Pred_. The percentage of posterior predictive draws of *T*_Pred_ that are greater than the anticipated completion date *T* is the trial’s estimated risk of accrual failure: *P*_Late_ =  *Pr* (*T*_Pred_ > *T*| Data)*.*

Overall, we will summarize and compare these two prediction outcomes. We will use different visualization methods to illustrate the differences between the Bayesian and the linear frequentist prediction models, and the comparison between *P*_late_ and the recruitment time of trial actually running.

This final step will bring together all the results from the analyses described under points 1–3 into a freely accessible tool. This tool should be user-friendly, and the parameters needed to provide estimates should be readily available for trial teams. The tool should help trialists to (1) estimate required time for completion of enrolment when planning the trial and (2) monitor and adjust planning while conducting the trial. For instance, if an investigator wants to estimate how long it will take to recruit 400 participants to a surgical RCT comparing two techniques for wound closure, it would be important to know how many sites were included in similar trials and which recruitment rate one can expect at each site. Also, the time lag between site activation and first patients enrolled would be important information. The tool could first provide clinical researchers with estimates based on recruitment data from similar surgical RCTs, preferably conducted in the same country (depending on our findings whether medical field and country are important predictors of recruitment time). With these estimates, one could then build a recruitment projection by adding sites and also by adding uncertainties to account for various issues related to the community (e.g., different degrees of interest and equipoise in the research question) or the center (e.g., different research personnel and capacity). Uncertainties could be modeled (e.g., + 10% time lag between approval and first patient enrolled). During the conduct of the trial, one could then step by step replace the initial estimates by real-world recruitment data from the trial to improve estimates on the time needed to recruit the required number of patients or to simulate how much time one could save if more sites were added. We will develop such a tool with input from recruitment experts and future users (trialists and trial coordinators), employ user testing [19, 20], and will also compare the tool’s usability and performance to another recently described web-based tool [21].

## Discussion

This project will address questions that clinical researchers, funding bodies, and research ethics committees are often confronted with: What are the chances that a specific trial achieves its recruitment target? How long will it take to recruit the target statistical sample size? When should researchers decide about changes in their recruitment strategy or about early trial discontinuation? The proposed project aims to provide reliable answers to these questions and an easy-to-apply tool for recruitment planning and monitoring in order to successfully complete RCTs and to allow for the most efficient allocation of scarce research resources. The current waste of valuable resources in clinical research is a huge international challenge [22, 23], and the need to increase efficiency of patient recruitment is a fundamental problem for trial investigators [5]. Trials that last longer than anticipated typically incur excessive management and trial unit resources and, importantly, delay the answer to research questions.

The strengths of our project include the fact that we use an empirical, international, comprehensive, and concerted approach to tackle the global practical challenge of reliably predicting and monitoring patient recruitment in RCTs. To the best of our knowledge, there is no other project comparable in size and scope. We will include a broad variety of recent RCTs increasing the external validity of our findings. By making our results, methods, and the resulting tool accessible through a dedicated website, we aim to further encourage other investigators sharing recruitment data to further optimize the model and broaden external validity.

Our project has several potential limitations. First, within our data collection, we do not capture the comprehensive planning and administrative process to set up an RCT on national or international level. These processes and speed of administration vary among countries (e.g., the time between ethical approval and the start of the trial). Therefore, this part of the planning phase cannot be part of our investigations and we cannot adjust for regional differences. Second, several RCTs with time to event outcomes (e.g., progression-free survival) are planned and powered based on the number of events observed over time and not a specific number of patients needed to be recruited [24]. Different models have been proposed to predict the occurrence of events of interest in a study population [18]. Based on our data, we cannot investigate this approach, because such time to event data are not available to us in this cohort of RCTs. Third, we anticipate that we will have only limited information on the time points when centers have been initiated and approved to recruit patients. As outlined previously, recruitment of centers for multicenter RCTs is also an important part of planning the recruitment process [7]. However, we anticipate that we can only investigate this issue of site recruitment in a sub-sample of RCTs where date of center activation is available. Fourth, we cannot systematically capture and consider the choices, rationale, or expertise of the investigators, staff, or the research network in conducting RCTs, which likely also affects the speed and ultimate success of the patient recruitment process. Finally, we do not have information on center characteristics that may also influence recruitment performance.

In summary, we will investigate the process of patient recruitment to reduce the risk of RCTs being discontinued for poor recruitment and produce a tool to support recruitment planning. We hope to contribute to the evidence base that will inform the conduct of clinical research, and enhance its efficiency by the comprehensive, concerted, international effort motivating this project.

## Trial status

Data collection of the study is still ongoing (started December 2016), and we anticipate finishing the project by end of 2021.

## Data Availability

Not applicable, because this manuscript does not include any data; however, we aim to make individual data available on publicly accessible data repositories after complete data collection and analyses have been finished and published.

## Abbreviations

ACTG: AIDS Clinical Trials Group
CTTI: Clinical Trials Transformation Initiative
EKNZ: Research ethics committee North West and Central Switzerland
KUCC: University of Kansas Cancer Center
MCMC: Markov-Chain Monte-Carlo
RCT: Randomized clinical trial
SAKK: Swiss Group for Clinical Cancer Research
